# Activation of Thioglycosides with Copper(II) Bromide

**DOI:** 10.3390/molecules27217354

**Published:** 2022-10-29

**Authors:** Faranak Pooladian, Samira Escopy, Alexei V. Demchenko

**Affiliations:** 1 Department of Chemistry, Saint Louis University, 3501 Laclede Ave, St. Louis, MO 63103, USA; 2 Department of Chemistry and Biochemistry, University of Missouri—St. Louis, One University Boulevard, St. Louis, MO 63121, USA

**Keywords:** glycosylation, synthesis, activation, oligosaccharides, transition metals

## Abstract

Reported herein is a new protocol for glycosidation of alkyl and aryl thioglycosides in the presence of copper(II) bromide. While the activation with CuBr_2_ alone was proven suitable for reactive glycosyl donors, the activation of less reactive donors was more efficient in the presence of triflic acid as an additive. A variety of thioglycoside donors in reactions with different glycosyl acceptors were investigated to determine the initial scope of this reaction.

## 1. Introduction

**Scheme 1:**
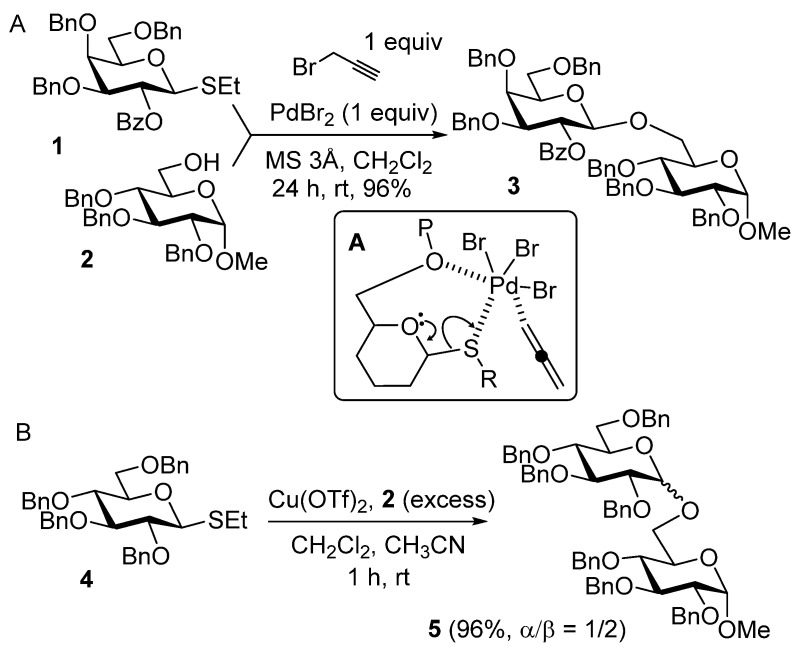
Previous examples of ethylthio glycoside activation in the presence of palladium (**A**) and copper salts (**B**).

A myriad of approaches have been developed for the chemical synthesis of glycosidic linkages [1,2,3]. Glycosyl halides [4,5], glycosyl imidates [6], and thioglycosides [7] have become the most prominent glycosyl donors utilized in chemical glycosylation and oligosaccharide synthesis. First synthesized by Fischer in 1909 [8], thioglycosides are stable towards a majority of protecting group manipulations albeit can be readily activated in the presence of mild thiophilic reagents [7,9,10,11,12]. Among a plethora of known thiophilic promoters, transition metals have been used for decades. In fact, the first known method for thioglycoside activation makes use of mercury(II) salts [13]. Nevertheless, only recently the activation of conventional thioglycosides through the direct coordination of a metal salt with the anomeric sulfur has been investigated in detail. First reported by Pohl et al., a sub-stoichiometric amount of Ph_3_Bi(OTf)_2_ was able to activate propylthio glycosides [14,15]. Subsequently, Sureshan et al. [16] performed activation of thioglycosides using a sub-stoichiometric amount of AuCl_3_. Zhu et al. [17] also showed that propargylthio glycosyl donors are activated through the direct coordination of Au(III) to the sulfur atom rather than the remote pathway via the alkyne functionality. As a part of our efforts toward the development of novel methods for glycosylation, previously we reported that alkyl/aryl thioglycosides can be activated with palladium(II) bromide (PdBr_2_) [18]. It has been found that the activation can be performed in the presence of PdBr_2_ only, but an additive (propargyl bromide) accelerates the activation process. A preliminary mechanistic analysis relying on ^1^H NMR spectroscopy revealed that propargyl bromide could form a more reactive reaction intermediate and possibly acts as the leaving group scavenger. For example, when thiogalactoside **1** was activated for glycosidation with glycosyl acceptor **2** in the presence of PdBr_2_ only, the reaction was slow and disaccharide **3** was obtained in 76% yield in 48 h. However, in the presence of propargyl bromide, disaccharide **3** was obtained in 96% yield within 24 h (Scheme 1A). It was postulated that the reaction proceeds via the intermediacy of complex **A**. In an effort to identify greener and more accessible transition metal salts, reported herein is our investigation of copper(II)-promoted activation of thioglycosides.

## 2. Results and Discussion

Our interest to copper(II) was sparked by a report by Dondoni, Marra, and Massi wherein the synthesis of disaccharides was achieved by the activation of ethylthio glycosides promoted by copper(II) triflate [19]. We were fascinated by these results because Cu(OTf)_2_ is a well-known activator for reactive glycosyl donors, such as halides^5^ or thioimidates [20,21,22], but not conventional alkyl/aryl thioglycosides. In accordance with the reported protocol, thioglycoside **4** was reacted with excess of acceptor **2** to afford the corresponding disaccharide **5** in a high yield albeit with poor stereoselectivity (Scheme 1B) [19].

By means of a personal communication with Professor Marra, the authors have learnt that the choice of the reaction solvent is essential. Marra and co-workers observed that the activation of thioglycosides promoted by Cu(OTf)_2_ proceeded much faster in acetonitrile (or MeCN/DCM), a solvent in which Cu(OTf)_2_ is highly soluble. The solvent was suspected to act as a ligand judged by the color change of the reaction mixture. Marra and co-workers have also suspected that reactions in MeCN proceeded through a single-electron transfer, whereas for reactions in DCM the copper salt acted as a thiophilic metal. It was also noted that these reaction conditions were better suited for the activation of “armed” glycosyl donors such as per-*O*-benzylated thioglycoside **4**. These observations were made in 2005, and to the best of our knowledge no follow-up investigation has yet emerged.

To elaborate on this discovery, for our own study we chose copper(II) bromide (cupric bromide) as a significantly cheaper and less moisture sensitive alternative to Cu(OTf)_2_. For the initial experimentation, we selected a highly reactive galactosyl donor **1 [23]** equipped with the superarming protecting group pattern [24], and a conventional primary glycosyl acceptor **2** [25]. When the reaction of glycosyl donor **1** with acceptor **2** was promoted with 0.8 equiv of CuBr_2_ in DCM at rt, disaccharide **3** [26] was obtained in 45% in 24 h (Table 1, entry 1). Complete 1,2-*trans* selectivity achieved in this reaction is due to the participation of the neighboring 2-*O*-benzoyl ester group, a well-known effect in carbohydrate chemistry [27]. Encouraged by this promising result, we performed activations in the presence of 1.5, 2.0, and 2.5 equiv of CuBr_2_. As a result, disaccharide **3** was obtained in 70, 75, and 96% yield, respectively (entries 2–4). Therefore, we chose to conduct all subsequent experiments in the presence of 2.5 equiv of the promoter.

These reactions were not swift and required 24 h to complete, and to enhance the reaction rates, we investigated the effect of the reaction temperature. When the reaction was performed at 40 °C, it was completed within 4 h. However, the shorter reaction time has also translated into a reduced yield of 84% for disaccharide **3** (entry 5). We then investigated the effect of the reaction solvent. When the reaction was carried out in 1,2-DCE at rt, disaccharide **3** was produced in an excellent yield of 98% and the reaction time was slightly reduced to 22 h (entry 6). Like in the case of reactions in DCM, increasing the reaction temperature (to 80 °C in this case) led to a decreased reaction time to 3 h, but also a decreased yield of 85% for disaccharide **3** (entry 7). Further investigation of the reaction solvent did not result in the anticipated gains. Although reactions in MeCN/1,2-DCE or neat MeCN were significantly faster at rt, 6 h or 1.5 h, the yields of disaccharide **3** were also reduced to 70 or 80%, respectively (entries 8 and 9). As a result of this optimization study, we identified reaction conditions listed in entry 6, CuBr_2_ (2.5 equiv) in 1,2-DCE at rt, as the most suitable conditions for the activation of donor **2**. It should be noted that other copper(II) salts such as Cu(OTf)_2_ or CuCl_2_ gave inferior results under these reaction conditions. Thus, disaccharide **3** was obtained in 24 or 16% yield, respectively, in 24 h (entries 10 and 11). An attempt to perform the activation in the presence of copper(I) bromide resulted in no reaction after 24 h (entry 12).

When the developed reaction conditions comprising 2.5 equiv CuBr_2_ in 1,2-DCE at rt were applied to the activation of per-*O*-benzoylated glycosyl donor **6** [28,29] for reaction with acceptor **2**, the corresponding disaccharide **7** [30] was obtained in a disappointing yield of 36% after 24 h (entry 13). This result was not totally unexpected due to a significantly lower reactivity of donor **6** due to its disarming protecting group pattern [31] (all esters) in comparison with the superarmed donor **1**. Hence, we conducted further optimization of reaction conditions to enhance glycosylations with donor **6**. This included varying all major factors, solvent, temperature, and additives, while keeping the amount of CuBr_2_ constant. Pilot investigation of the reaction solvent and temperature led to only marginal improvements. Thus, reaction in MeCN produced disaccharide **7** in 50% yield in 6 h (entry 14). Reaction in 1,2-DCE performed at 80 ^o^C led to the formation of **7** in 70% in 4 h (entry 15). The desired solution was found by implementing TfOH as an additive for this reaction, and the optimal amount was found to be 0.5 equiv. Thus, glycosidation of donor **6** with acceptor **2** in the presence of CuBr_2_ (2.5 equiv) and TfOH (0.5 equiv) in DCE at rt led to the formation of disaccharide **7** in 96% yield in 24 h (entry 16)! A control experiment performed with the reactive glycosyl donor **1** in the presence of CuBr_2_ (2.5 equiv) and TfOH (0.5 equiv) in DCE at rt led to disaccharide **3** in a reduced yield of 73% (entry 17). This result was rationalized by the enhanced rate of hydrolysis of the glycosyl donor that is taking place in the presence of TfOH (see the SI for NMR monitoring experiments). Also observed was that in the absence of glycosyl acceptor, the glycosyl bromide will be slowly produced in the presence of CuBr_2_ alone. Traces were present between 4 and 20 h. In the presence of CuBr_2_ and TfOH, hemiacetal, the product of donor hydrolysis is produced first (2–4 h), and then slow production of the glycosyl bromide begins (20 h).

Therefore, as a result of these optimization studies, we concluded that reactive glycosyl donors are best activated in the presence of CuBr_2_ (2.5 equiv) only, whereas unreactive donors may require TfOH (0.5 equiv) as an additive. With these considerations in mind, we applied the developed reaction conditions to glycosidation of acceptor **2** with a variety of thioglycosides of different sugar series equipped with various protecting and leaving groups. All reactions have been performed using Conditions A (CuBr_2_ only) and the least reactive glycosyl donors have also been activated using Conditions B (CuBr_2_/TfOH). This study is summarized in Table 2.

We first investigated a series of other galactosyl donors; and these reactions showed a decline in the product yields that was clearly correlated with the predicted general trends for relative reactivity of glycosyl donors [32,33,34,35,36,37,38,39]. Per-*O*-benzylated galactosyl donor **8** [40,41] produced the corresponding disaccharide **9** [42] in 72% yield as a mixture of anomers (*α/β* = 1/1.5, entry 1, Table 2). A similar reaction outcome was achieved with α-thioglycoside **10** [43] and disaccharide **9** was obtained in 61% (*α/β* = 1/1.8, entry 2). The lower yield was rationalized by lower reactivity of α-thioglycosides than their β-linked counterparts [37]. Glycosidation of phenylthio glycoside **11** [44] produced disaccharide **9** in 45% yield (*α/β* = 1/1.5, entry 3). This lower yield was rationalized by general lower reactivity of arylthio glycosides versus their alkylthio counterparts [45]. Glycosylation of partially acylated donor **12** [46] using conditions A produced disaccharide **13** [47] in a modest yield of 35% (entry 4). Expectedly, this result with partially disarmed glycosyl donor **12** could be enhanced when the reaction was repeated using Conditions B. In this case, disaccharide **13** was obtained in an improved yield of 54% (entry 4). These reactions were relatively α-selective [47], *α/β* = 6.5/1 for both Conditions A and Conditions B. A similar enhancement of stereoselectivity was recently observed for 3,4-di-*O*-benzoylated galactosyl donors [48].

We then investigated a series of glucosyl donors, which provided comparable results under reaction conditions A. Thus, per-*O*-benzylated ethyl, tolyl, and phenyl thioglycosides **4** [49], **15** [50], and **16** [51] produced disaccharide **14** [42] in 60–82% yield (*α/β* from 1.1/1 to 1/1.4, entries 5–7). Similarly to that observed in the D-galacto series, SPh donor was the least reactive in this case. 6-O-TBDPS-protected donor **17 [52]** produced disaccharide **18** [53] in 73% yield (*α/β* = 2.0/1, entry 8). Even per-*O*-benzoylated donor **19** [29] reacted well under conditions A and the corresponding disaccharide **20** [54] was isolated in 70% yield (entry 9). Only one example in this series wherein torsionally deactivated [55,56] glycosyl donor **21** have failed to produce good yields. Thus, under conditions A or B, disaccharide **22** [57] was obtained in 15 or 28% yield, respectively (*α/β* = 3.1/1 in both cases, entry 10). Glycosidation of glucosamine-derived donor **23** [58] afforded disaccharide **24** [18] in a respectable yield of 76% (entry 11). Glycosidation of per-benzylated mannosyl donor **25** [59] was also possible, and the corresponding disaccharide **26** [60,61] was obtained in 60% yield with no selectivity (entry 12).

We then turned our attention on investigating glycosylation of other glycosyl acceptors using glycosyl donor **1** under conditions A. Glycosylation of secondary glycosyl acceptors **27** [25] or **29** [25] was very effective, and the respective disaccharides **28** [18] and **30** [26] were obtained in 93–95% yield (entries 1 and 2, Table 3). Glycosylation of hindered 4-OH acceptor **31** [25] was less efficient, nevertheless disaccharide **32** [26] was obtained in a respectable yield of 70% (entry 1). Finally, glycosylation of electronically deactivated benzoylated 6-OH acceptor **33** [62] produced disaccharide **34** [63] in 95% yield (entry 4).

## 3. Experimental

General. All chemicals used were reagent grade and used as supplied. The ACS grade solvents used for reactions were purified and dried in accordance with standard procedures. Column chromatography was performed on silica gel 60 (70–230 mesh), reactions were monitored by TLC on Kieselgel 60 F_254_. The compounds were detected by examination under UV light and by charring with 10% sulfuric acid in methanol. Solvents were removed under reduced pressure at <40 °C. CH_2_Cl_2_ and ClCH_2_CH_2_Cl were distilled from CaH_2_ directly prior to the application. Molecular sieves (3 Å), used for reactions, were crushed and activated in *vacuo* at 390 °C during 8 h in the first instance and then for 2–3 h at 390 °C directly prior to application. ^1^H NMR spectra were recorded at 400 MHz, ^13^C NMR spectra were recorded at 100 MHz. The ^1^H NMR chemical shifts are referenced to tetramethylsilane (δ_H_ = 0 ppm) or CHCl_3_ (δ_H_ = 7.26 ppm) for ^1^H NMR spectra for solutions in CDCl_3_. Anomeric ratios (if applicable) were determined by comparison of the integral intensities of relevant signals in ^1^H NMR spectra (see the Supplementary Material).

### 3.1. Synthesis of Building Blocks

Ethyl 2-*O*-benzoyl-3,4,6-tri-*O*-benzyl-1-thio-*β*-D-galactopyranoside (1) was synthesized as reported previously and its analytical data was in accordance with that previously described [23].

Methyl 2,3,4-tri-*O*-benzyl-*α*-D-glucopyranoside (2) was synthesized as reported previously and its analytical data was in accordance with that previously described [25].

Ethyl 2,3,4,6-tetra-*O*-benzyl-1-thio-*β*-D-glucopyranoside (4) was synthesized as reported previously and its analytical data was in accordance with that previously described [49].

Ethyl 2,3,4,6-tetra-*O*-benzoyl-1-thio-*β*-D-galactopyranoside (6) was synthesized as reported previously and its analytical data was in accordance with that previously described [28,29].

Ethyl 2,3,4,6-tetra-*O*-benzyl-1-thio-*β*-D-galactopyranoside (8) was synthesized as reported previously and its analytical data was in accordance with that previously described [40,41].

Ethyl 2,3,4,6-tetra-*O*-benzyl-1-thio-*α*-D-galactopyranoside (10) was synthesized as reported previously and its analytical data was in accordance with that previously described [43].

Phenyl 2,3,4,6-tetra-*O*-benzyl-1-thio-β-D-galactopyranoside (11) was synthesized as reported previously and its analytical data was in accordance with that previously described [44].

Ethyl 3,4-di-*O*-acetyl-2,6-di-*O*-benzyl-1-thio-β-D-galactopyranoside (12) was synthesized as reported previously and its analytical data was in accordance with that previously described [46].

Tolyl 2,3,4,6-tetra-*O*-benzyl-1-thio-*β*-D-glucopyranoside (15) was synthesized as reported previously and its analytical data was in accordance with that previously described [50,51].

Phenyl 2,3,4,6-tetra-*O*-benzyl-1-thio-β-D-glucopyranoside (16) was synthesized as reported previously and its analytical data was in accordance with that previously described [51].

Ethyl 2,3,4-tri-*O*-benzyl-6-*O-tert*-butyldiphenylsilyl-1-thio-β-D-glucopyranoside (17) was synthesized as reported previously and its analytical data was in accordance with that previously described [52].

Ethyl 2,3,4,6-tetra-*O*-benzoyl-1-thio-*β*-D-glucopyranoside (19) was synthesized as reported previously and its analytical data was in accordance with that previously described [29].

Ethyl 2,3-di-*O*-benzyl-4,6-*O*-benzylidene-1-thio-β-D-glucopyranoside (21) was synthesized as reported previously and its analytical data was in accordance with that previously described [64].

Ethyl 4,6-di-*O*-benzyl-2-deoxy-3-*O*-fluorenylmethoxycarbonyl-2-phthalimido-1-thio-*β*-D-glucopyranoside (23) was synthesized as reported previously and its analytical data was in accordance with that previously described [58].

Ethyl 2,3,4,6-tetra-*O*-benzyl-1-thio-α-D-mannopyranoside (25) was synthesized as reported previously and its analytical data was in accordance with that previously described [59].

Methyl 3,4,6-tri-*O*-benzyl-α-D-glucopyranoside (27) was synthesized as reported previously and its analytical data was in accordance with that previously described [25].

Methyl 2,4,6-tri-*O*-benzyl-*α*-D-glucopyranoside (29) was synthesized as reported previously and its analytical data was in accordance with that previously described [25,65].

Methyl 2,3,6-tri-*O*-benzyl-α-D-glucopyranoside (31) was synthesized as reported previously and its analytical data was in accordance with that previously described [25].

Methyl 2,3,4-tri-*O*-benzoyl-*α*-D-glucopyranoside (33) was synthesized as reported previously and its analytical data was in accordance with that previously described [62].

### 3.2. Synthesis of Disaccharides

#### 3.2.1. Method A—General Procedure for Glycosidation of Thioglycosides in the Presence of CuBr_2_

A mixture containing thioglycoside donor (30 mg, 0.040–0.050 mmol), glycosyl acceptor (0.030–0.040 mmol), and freshly activated molecular sieves (3 Å, 90 mg) in dry 1,2-dichloroethane (C_2_H_4_Cl_2_, DCE, 1.0 mL) was stirred under argon for 1 h at rt. After that, copper bromide (CuBr_2_, 2.5 equiv to donor, 0.100–0.125 mmol) was added and the reaction mixture was stirred for 24 h at rt. The solids were filtered off through a pad of Celite and rinsed successively with DCM. The combined filtrate (~20 mL) was washed with H_2_O (2 × 5 mL). The organic phase was separated, dried with MgSO_4_, and concentrated under reduced pressure. The residue was purified by column chromatography on silica gel (ethyl acetate–hexanes gradient elution) to afford a disaccharide derivative in yields listed in tables and below.

#### 3.2.2. Method B—General Procedure for Glycosidation of Thioglycosides in the Presence of CuBr_2_ and TfOH

A mixture containing thioglycoside donor (30 mg, 0.040–0.050 mmol), glycosyl acceptor (0.030–0.040 mmol), and freshly activated molecular sieves (3 Å, 90 mg) in dry 1,2-dichloroethane (C_2_H_4_Cl_2_, DCE, 1.0 mL) was stirred under argon for 1 h at rt. After that, copper bromide (CuBr_2_, 2.5 equiv to donor, 0.100–0.125 mmol) and TfOH (0.50 equiv to donor, 0.020–0.025) was added and the reaction mixture was stirred for 24 h at rt. The solids were filtered off through a pad of Celite and rinsed successively with DCM. The combined filtrate (~20 mL) was washed with H_2_O (2 × 5 mL). The organic phase was separated, dried with MgSO_4_, and concentrated under reduced pressure. The residue was purified by column chromatography on silica gel (ethyl acetate–hexanes gradient elution) to afford a disaccharide derivative in yields listed in tables and below.

Methyl 6-*O*-(2-*O*-benzoyl-3,4,6-tri-*O*-benzyl-*β*-D-galactopyranosyl)-2,3,4-tri-*O*-benzyl-*α*-D-glucopyranoside (3) was obtained from thioglycoside 1 and glycosyl acceptor 2 by Method A in up to 98% yield as a clear film. Analytical data for 3 was in accordance with that reported previously [26].

Methyl 6-*O*-(2,3,4,6-tetra-*O*-benzoyl-β-D-galactopyranosyl)-2,3,4-tri-*O*-benzyl-α-D-glucopyranoside (7) was obtained from thioglycoside 6 and glycosyl acceptor 2 by Method B in up to 96% yield as a white amorphous solid. Analytical data for 7 was in accordance with that reported previously [30].

Methyl 2,3,4-tri-*O*-benzyl-6-*O*-(2,3,4,6-tetra-*O*-benzyl-*α/β*-D-galactopyranosyl)-*α*-D-glucopyranoside (9) was obtained from thioglycosides 8, 10 or 11 and glycosyl acceptor 2 by Method A in 72, 62, or 45% yield, respectively (*α/β* = 1/1.5–1.8) as a colorless syrup. Analytical data for 9 was in accordance with that reported previously [42].

Methyl 6-*O*-(3,4-di-*O*-acetyl-2,6-di-*O*-benzyl-α/β-D-galactopyranosyl)-2,3,4-tri-*O*-benzyl-α-D-glucopyranoside (13) was obtained from thioglycoside 12 and glycosyl acceptor 2 by Method A or Method B in 35 or 54% yield, respectively (*α/β* = 6.5/1) as a colorless syrup. Analytical data for 13 was in accordance with that reported previously [47].

Methyl 2,3,4-tri-*O*-benzyl-6-*O*-(2,3,4,6-tetra-*O*-benzyl-*α/β*-D-glucopyranosyl)-*α*-D-glucopyranoside (14) was obtained from thioglycosides 4, 15 or 16 and glycosyl acceptor 2 by Method A in 82, 79 or 60% yield, respectively (*α/β* = from 1.1/1 to 1/1.4) as a colorless syrup. Analytical data for 14 was in accordance with that reported previously [42].

Methyl 2,3,4-tri-*O*-benzyl-6-*O*-(2,3,4-tri-*O*-benzyl-6-*O-tert*-butyldiphenylsilyl-α/β-D-glucopyranosyl)-α-D-glucopyranoside (18) was obtained from thioglycoside 17 and glycosyl acceptor 2 by Method A in 73% yield (*α/β* = 2.0/1) as a colorless syrup. Analytical data for 18 was in accordance with that reported previously [53].

Methyl 6-*O*-(2,3,4,6-tetra-*O*-benzoyl-*β*-D-glucopyranosyl)-2,3,4-tri-*O*-benzyl-*α*-D-glucopyranoside (20) was obtained from thioglycoside 19 and glycosyl acceptor 2 by Method A in 70% yield as a clear film. Analytical data for 20 was in accordance with that reported previously [54].

Methyl 2,3,4-tri-*O*-benzyl-6-*O*-(2,3-di-*O*-benzyl-4,6-*O*-benzylidene-α/β-D-glucopyranosyl)-α-D-glucopyranoside (22) was obtained from thioglycoside 21 and glycosyl acceptor 2 by Method A or Method B in 15 or 28% yield, respectively (*α/β* = 3.1/1) as a colorless syrup. Analytical data for 22 was in accordance with that reported previously [57].

Methyl 2,3,4-tri-*O*-benzyl-6-*O*-(4,6-di-*O*-benzyl-2-deoxy-3-*O*-fluorenylmethoxycarbonyl-2-phthalimido-*β*-D-glucopyranosyl)-*α*-D-glucopyranoside (24) was obtained from thioglycoside 23 and glycosyl acceptor 2 by Method A in 76% yield as a clear film. Analytical data for 24 was in accordance with that reported previously [18].

Methyl 2,3,4-tri-*O*-benzyl-6-*O*-(2,3,4,6-tetra-*O*-benzyl-α/β-D-mannopyranosyl)-α-D-glucopyranoside (26) was obtained from thioglycoside 25 and glycosyl acceptor 2 by Method A in 60% yield (*α/β* = 1/1.1) as a colorless syrup. Analytical data for 26 was in accordance with that reported previously [60,61].

Methyl 2-*O*-(2-*O*-benzoyl-3,4,6-tri-*O*-benzyl-β-D-galactopyranosyl)-3,4,6-tri-*O*-benzyl-α-D-glucopyranoside (28) was obtained from thioglycoside 1 and glycosyl acceptor 27 by Method A in 93% yield as a colorless syrup. Analytical data for 28 was in accordance with that reported previously [18].

Methyl 3-*O*-(2-*O*-benzoyl-3,4,6-tri-*O*-benzyl-*β-*D-galactopyranosyl)-2,4,6-tri-*O*-benzyl-*α-*D-glucopyranoside (30) was obtained from thioglycoside 1 and glycosyl acceptor 29 by Method A in 95% yield as a clear film. Analytical data for 30 was in accordance with that reported previously [26].

Methyl 4-*O*-(2-*O*-benzoyl-3,4,6-tri-*O*-benzyl-β-D-galactopyranosyl)-2,3,6-tri-*O*-benzyl-α-D-glucopyranoside (32) was obtained from thioglycoside 1 and glycosyl acceptor 31 by Method A in 70% yield as a clear film. Analytical data for 32 was in accordance with that reported previously [26].

Methyl 2,3,4-tri-*O*-benzoyl-6-*O*-(2-*O*-benzoyl-3,4,6-tri-*O*-benzyl-*β*-D-galactopyranosyl)-*α*-D-glucopyranoside (34) was obtained from thioglycoside 1 and glycosyl acceptor 33 by Method A in 95% yield as a white amorphous solid. Analytical data for 34 was in accordance with that reported previously [63].

## 4. Conclusions

A new method for the activation of thioglycosides has been developed. The activation with CuBr_2_ can be sluggish with unreactive thioglycoside donors. In these cases, the outcome can be improved in the presence of triflic acid additive. Upon standardizing the basic reaction conditions for both reactive and unreactive glycosyl donors, further examination of various thioglycosides has been performed. In most cases, our activation system was effective and predictable, but the product yields were largely dependent on the reactivity of the glycosyl donor. We believe that this study will fundamentally contribute to other developments of copper-catalyzed reactions with carbohydrates [66,67,68,69,70,71,72,73,74,75]. Further optimization of the reaction conditions, investigation of the reaction mechanism, and its application in automated synthesis of glycans are currently underway in our laboratory.

## Figures and Tables

**Table 1:** CuBr_2_-mediated glycosidation of donors **1** and **6** with glycosyl acceptor **2**.

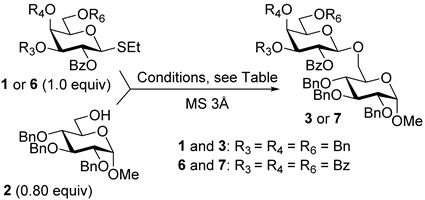			
Entry	Donor	Conditions	Product, Yield
1	**1**	CuBr_2_ (0.8 equiv), DCM, rt, 24 h	**3**, 45%
2	**1**	CuBr_2_ (1.5 equiv), DCM, rt, 24 h	**3**, 70%
3	**1**	CuBr_2_ (2.0 equiv), DCM, rt, 24 h	**3**, 75%
4	**1**	CuBr_2_ (2.5 equiv), DCM, rt, 24 h	**3**, 96%
5	**1**	CuBr_2_ (2.5 equiv), DCM, 40 °C, 4 h	**3**, 84%
6	**1**	CuBr_2_ (2.5 equiv), DCE, rt, 22 h	**3**, 98%
7	**1**	CuBr_2_ (2.5 equiv), DCE, 80 °C, 3 h	**3**, 85%
8	**1**	CuBr_2_ (2.5 equiv), DCE/MeCN (1/1, *v*/*v*), rt, 6 h	**3**, 70%
9	**1**	CuBr_2_ (2.5 equiv), MeCN, rt, 1.5 h	**3**, 80%
10	**1**	Cu(OTf)_2_ (2.5 equiv), DCE, rt, 24 h	**3**, 24%
11	**1**	CuCl_2_ (2.5 equiv), DCE, rt, 24 h	**3**, 16%
12	**1**	CuBr (2.5 equiv), DCE, rt, 24 h	NR
13	**6**	CuBr_2_ (2.5 equiv), DCE, rt, 24 h	**7**, 36%
14	**6**	CuBr_2_ (2.5 equiv), MeCN, rt, 6 h	**7**, 50%
15	**6**	CuBr_2_ (2.5 equiv), DCE, 80 °C, 4 h	**7**, 70%
16	**6**	CuBr_2_ (2.5 equiv), TfOH (0.5 equiv), DCE, rt, 24 h	**7**, 96%
17	**1**	CuBr_2_ (2.5 equiv), TfOH (0.5 equiv), DCE, rt, 3 h	**3**, 73%

**Table 2:** Broadening the scope of the CuBr_2_-assisted glycosylation to other glycosyl donors.

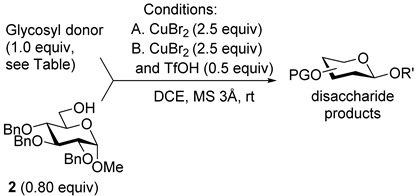			
Entry	Donor	Conditions	Product, Yield, Ratio *α/β*
1	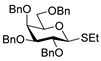 **8**	A	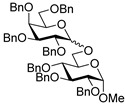 **9**, 72%, *α/β* = 1/1.5
2	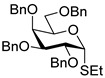 **10**	A	**9**, 61%, *α/β* = 1/1.8
3	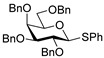 **11**	A	**9**, 45%, *α/β* = 1/1.5
4	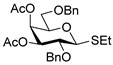 **12**	A or B	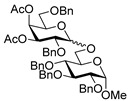 **13**, 35%, *α/β* = 6.5/1 or 54%, *α/β* = 6.5/1
5	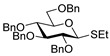 **4**	A	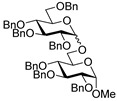 **14**, 82%, *α/β* = 1.1/1
6	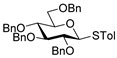 **15**	A	**14**, 79%, *α/β* = 1/1.4
7	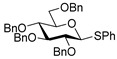 **16**	A	**14**, 60%, *α/β* = 1/1.1
8	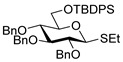 **17**	A	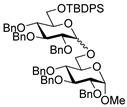 **18**, 73%, *α/β* = 2.0/1
9	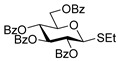 **19**	A	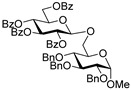 **20**, 70%, β only
10	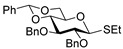 **21**	A or B	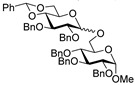 **22**, 15%, α/β = 3.1/1 or 28%, α/β = 3.1/1
11	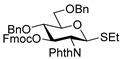 **23**	A	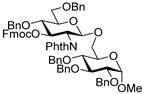 **24**, 76%, β only
12	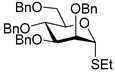 **25**	A	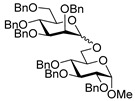 **26**, 60%, *α/β* = 1/1.1

**Table 3:** Extending the scope of the CuBr_2_-promoted glycosylation to other glycosyl acceptors.

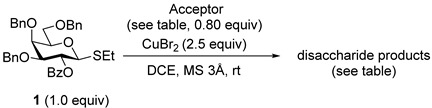		
Entry	Acceptor	Product, Yield
1	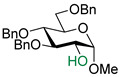 **27**	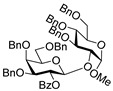 **28**, 93%
2	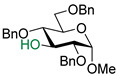 **29**	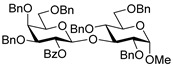 **30**, 95%
3	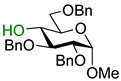 **31**	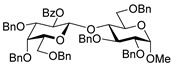 **32**, 70%
4	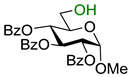 **33**	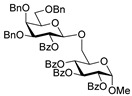 **34**, 95%

## Supplementary Materials

The following supporting information can be downloaded at: https://www.mdpi.com/article/10.3390/molecules27217354/s1, Figure S1: NMR Monitoring to Understand Mechanistic Aspects of the Reaction; Figure S2: ^1^H NMR Sectra of Known Compounds; Figure S3: ^1^H NMR (CDCl3 400 MHz) of compound **3**; Figure S4: ^1^H NMR (CDCl3 400 MHz) of compound **7**; Figure S5: ^1^H NMR (CDCl3 400 MHz) of compound **9**; Figure S6: ^1^H NMR (CDCl3 400 MHz) of compound **13**; Figure S7: ^1^H NMR (CDCl3 400 MHz) of compound **14**; Figure S8: ^1^H NMR (CDCl3 400 MHz) of compound **18**; Figure S9: ^1^H NMR (CDCl3 400 MHz) of compound **20**; Figure S10: ^1^H NMR (CDCl3 400 MHz) of compound **22**; Figure S11: ^1^H NMR (CDCl3 400 MHz) of compound **24**; Figure S12: ^1^H NMR (CDCl3 400 MHz) of compound **26**; Figure S13: ^1^H NMR (CDCl3 400 MHz) of compound **28**; Figure S14: ^1^H NMR (CDCl3 400 MHz) of compound **30**; Figure S15: ^1^H NMR (CDCl3 400 MHz) of compound **32**; Figure S16: ^1^H NMR (CDCl3 400 MHz) of compound **34**.

## Sample Availability

Samples of all compounds are available from the authors upon request.

## References

[1] Demchenko A.V. (2008). Handbook of Chemical Glycosylation: Advances in Stereoselectivity and Therapeutic Relevance.

[2] Zhu X., Schmidt R.R. (2009). New principles for glycoside-bond formation. Angew. Chem. Int. Ed..

[3] Panza M., Pistorio S.G., Stine K.J., Demchenko A.V. (2018). Automated chemical oligosaccharide synthesis: Novel approach to traditional challenges. Chem. Rev..

[4] Kulkarni S.S., Gervay-Hague J., Demchenko A.V. (2008). Glycosyl chlorides, bromides and iodides. Handbook of Chemical Glycosylation.

[5] Singh Y., Geringer S., Demchenko A.V. (2022). Synthesis and glycosidation of anomeric halides: Evolution from early studies to modern methods of the 21st century. Chem. Rev..

[6] Zhu X., Schmidt R.R., Demchenko A.V. (2008). Glycoside synthesis from 1-oxygen-substituted glycosyl imidates. Handbook of Chemical Glycosylation.

[7] Zhong W., Boons G.-J., Demchenko A.V. (2008). Glycoside synthesis from 1-sulfur/selenium-substituted derivatives: Thioglycosides in oligosaccharide synthesis. Handbook of Chemical Glycosylation.

[8] Fischer E., Delbruck K. (1909). Uber Thiophenol-glucoside. Ber. Dtsch. Chem. Ges..

[9] Oscarson S., Ernst B., Hart G.W., Sinay P. (2000). Thioglycosides. Carbohydrates in Chemistry and Biology.

[10] Codee J.D.C., Litjens R.E.J.N., van den Bos L.J., Overkleeft H.S., van der Marel G.A. (2005). Thioglycosides in sequential glycosylation strategies. Chem. Soc. Rev..

[11] Lian G., Zhang X., Yu B. (2015). Thioglycosides in Carbohydrate research. Carbohydr. Res..

[12] Escopy S., Demchenko A.V. (2022). Transition-Metal-Mediated Glycosylation with Thioglycosides. Chem. Eur. J..

[13] Ferrier R.J., Hay R.W., Vethaviyasar N. (1973). A potentially versatile synthesis of glycosides. Carbohydr. Res..

[14] Goswami M., Ellern A., Pohl N.L. (2013). Bismuth(V)-mediated thioglycoside activation. Angew. Chem. Int. Ed. Engl..

[15] Goswami M., Ashley D.C., Baik M.H., Pohl N.L. (2016). Mechanistic Studies of Bismuth(V)-Mediated Thioglycoside Activation Reveal Differential Reactivity of Anomers. J. Org. Chem..

[16] Vibhute A.M., Dhaka A., Athiyarath V., Sureshan K.M. (2016). A versatile glycosylation strategy via Au (III) catalyzed activation of thioglycoside donors. Chem. Sci..

[17] Adhikari S., Baryal K.N., Zhu D., Li X., Zhu J. (2013). Gold-catalyzed synthesis of 2-deoxy glycosides using S-but-3-ynyl thioglycoside donors. ACS Catal..

[18] Escopy S., Singh Y., Demchenko A.V. (2021). Palladium(II)-assisted activation of thioglycosides. Org. Biomol. Chem..

[19] Dondoni A., Marra A., Massi A. (2005). Hybrid solution/solid-phase synthesis of oligosaccharides by using trichloroacetyl isocyanate as sequestration-enabling reagent of sugar alcohols. Angew. Chem. Int. Ed..

[20] Pornsuriyasak P., Kamat M.N., Demchenko A.V. (2007). Glycosyl thioimidates as versatile glycosyl donors for stereoselective glycosylation and convergent oligosaccharide synthesis. ACS Symp. Ser..

[21] Szeja W., Grynkiewicz G., Demchenko A.V. (2008). Xanthates, thioimidates and other thio derivatives. Handbook of Chemical Glycosylation.

[22] Hasty S.J., Demchenko A.V. (2012). Glycosyl thioimidates as versatile building blocks for organic synthesis. Chem. Heterocycl. Compd..

[23] Grube M., Lee B.-Y., Garg M., Michel D., Vilotijević I., Malik A., Seeberger P.H., Varón Silva D. (2018). Synthesis of Galactosylated Glycosylphosphatidylinositol Derivatives from Trypanosoma brucei. Chem. Eur. J..

[24] Premathilake H.D., Mydock L.K., Demchenko A.V. (2010). Superarming common glycosyl donors by simple 2-O-benzoyl-3,4,6-tri-O-benzyl protection. J. Org. Chem..

[25] Ranade S.C., Kaeothip S., Demchenko A.V. (2010). Glycosyl alkoxythioimidates as complementary building blocks for chemical glycosylation. Org. Lett..

[26] Mukaiyama T., Takeuchi K., Jona H., Maeshima H., Saitoh T. (2000). A catalytic and stereoselective glycosylation with b-glycosyl fluorides. Helv. Chim. Acta.

[27] Goodman L. (1967). Neighboring-group participation in sugars. Adv. Carbohydr. Chem. Biochem..

[28] Dondoni A., Marra A., Scherrmann M.-C., Casnati A., Sansone F., Ungaro R. (1997). Synthesis and properties of O-glycosyl calix[4]arenes (calixsugars). Chem. Eur. J..

[29] Sail D., Kovac P. (2012). Benzoylated ethyl 1-thioglycosides: Direct preparation from per-O-benzoylated sugars. Carbohydr. Res..

[30] Nigudkar S.S., Parameswar A.R., Pornsuriyasak P., Stine K.J., Demchenko A.V. (2013). O-Benzoxazolyl imidates as versatile glycosyl donors for chemical glycosylation. Org. Biomol. Chem..

[31] Bandara M.D., Yasomanee J.P., Demchenko A.V., Bennett C.S. (2017). Application of armed, disarmed, superarmed and superdisarmed building blocks in stereocontrolled glycosylation and expeditious oligosaccharide synthesis. Selective Glycosylations: Synthetic Methods and Catalysts.

[32] Grice P., Ley S.V., Pietruszka J., Priepke H.W.M., Walther E.P.E. (1995). Tuning the reactivity of glycosides: Efficient one-pot oligosaccharide synthesis. Synlett.

[33] Douglas N.L., Ley S.V., Lucking U., Warriner S.L. (1998). Tuning glycoside reactivity: New tool for efficient oligosaccharides synthesis. J. Chem. Soc. Perkin Trans. 1.

[34] Zhang Z., Ollmann I.R., Ye X.S., Wischnat R., Baasov T., Wong C.H. (1999). Programmable one-pot oligosaccharide synthesis. J. Am. Chem. Soc..

[35] Green L.G., Ley S.V., Ernst B., Hart G.W., Sinay P. (2000). Protecting groups: Effects on reactivity, glycosylation specificity and coupling efficiency. Carbohydrates in Chemistry and Biology.

[36] Fraser-Reid B., Lopez J.C. (2011). . Reactivity Tuning in Oligosaccharide Assembly.

[37] Kaeothip S., Yasomanee J.P., Demchenko A.V. (2012). Glycosidation of thioglycosides in the presence of bromine: Mechanism, reactivity, and stereoselectivity. J. Org. Chem..

[38] Smith R., Muller-Bunz H., Zhu X. (2016). Investigation of alpha-Thioglycoside Donors: Reactivity Studies toward Configuration-Controlled Orthogonal Activation in One-Pot Systems. Org. Lett..

[39] Verma N., Tu Z., Lu M.S., Liu S.H., Renata S., Phang R., Liu P.K., Ghosh B., Lin C.H. (2021). Threshold of Thioglycoside Reactivity Difference Is Critical for Efficient Synthesis of Type I Oligosaccharides by Chemoselective Glycosylation. J. Org. Chem..

[40] Kihlberg J.O., Leigh D.A., Bundle D.R. (1990). The in situ activation of thioglycosides with bromine: An improved glycosylation method. J. Org. Chem..

[41] Chatterjee S., Moon S., Hentschel F., Gilmore K., Seeberger P.H. (2018). An Empirical Understanding of the Glycosylation Reaction. J. Am. Chem. Soc..

[42] Nguyen H.M., Chen Y.N., Duron S.G., Gin D.Y. (2001). Sulfide-mediated dehydrative glycosylation. J. Am. Chem. Soc..

[43] Kasbeck L., Kessler H. (1997). Convenient Syntheses of 2,3,4,6-Tetra-O-alkylated D-Glucose and D-Galactose. Liebigs Ann./Recl..

[44] Heuckendorff M., Poulsen L.T., Jensen H.H. (2016). Remote Electronic Effects by Ether Protecting Groups Fine-Tune Glycosyl Donor Reactivity. J. Org. Chem..

[45] Kaeothip S., Demchenko A.V. (2011). On orthogonal and selective activation of glycosyl thioimidates and thioglycosides: Application to oligosaccharide assembly. J. Org. Chem..

[46] Liu L., Bytheway I., Karoli T., Fairweather J.K., Cochran S., Li C., Ferro V. (2008). Design, synthesis, FGF-1 binding, and molecular modeling studies of conformationally flexible heparin mimetic disaccharides. Bioorg. Med. Chem. Lett..

[47] Li Z., Zhu L., Kalikanda J. (2011). Development of a highly α-selective galactopyranosyl donor based on a rational design. Tetrahedron Lett..

[48] Shadrick M., Singh Y., Demchenko A.V. (2020). Stereocontrolled α-galactosylation under cooperative catalysis. J. Org. Chem..

[49] Andersson F., Fugedi P., Garegg P.J., Nashed M. (1986). Synthesis of 1,2-cis-linked glycosides using dimethyl(methylthio)sulfonium triflate as promoter and thioglycosides as glycosyl donors. Tetrahedron Lett..

[50] Veleti S.K., Lindenberger J.J., Thanna S., Ronning D.R., Sucheck S.J. (2014). Synthesis of a Poly-hydroxypyrolidine-Based inhibitor of Mycobacterium tuberculosis GlgE. J. Org. Chem..

[51] France R.R., Rees N.V., Wadhawman J.D., Fairbanks A.J., Compton R.G. (2004). Selective activation of glycosyl donors using electrochemical techniques: A study of the thermodynamic oxidation potentials of a range of chalcoglycosides. Org. Biomol. Chem..

[52] Houdier S., Vottero P.J.A. (1994). Synthesis of benzylated cycloisomaltotri- and hexaosides. Angew. Chem. Int. Ed. Engl..

[53] Shaw M., Thakur R., Kumar A. (2019). Gold(III)-Catalyzed Glycosylation using Phenylpropiolate Glycosides: Phenylpropiolic Acid, An Easily Separable and Reusable Leaving Group. J. Org. Chem..

[54] Garcia B.A., Gin D.Y. (2000). Dehydrative glycosylation with activated diphenyl sulfonium reagents. Scope, mode of C(1)-hemiacetal activation, and detection of reactive glycosyl intermediates. J. Am. Chem. Soc..

[55] Fraser-Reid B., Wu Z., Andrews C.W., Skowronski E. (1991). Torsional effects in glycoside reactivity: Saccharide couplings mediated by acetal protecting groups. J. Am. Chem. Soc..

[56] Jensen H.H., Nordstrom L.U., Bols M. (2004). The disarming effect of the 4,6-acetal group on glycoside reactivity: Torsional or electronic?. J. Am. Chem. Soc..

[57] Ma X., Zheng Z., Fu Y., Zhu X., Liu P., Zhang L. (2021). A "Traceless" Directing Group Enables Catalytic SN2 Glycosylation toward 1,2-cis-Glycopyranosides. J. Am. Chem. Soc..

[58] Bandara M.D., Stine K.J., Demchenko A.V. (2019). The chemical synthesis of human milk oligosaccharides: Lacto-N-tetraose (Galβ1-3GlcNAcβ1-3Galβ1-4Glc). Carbohydr. Res..

[59] Wang C., Sanders B., Baker D.C. (2011). Synthesis of a glycodendrimer incorporating multiple mannosides on a glucoside core. Can. J. Chem..

[60] Hotha S., Kashyap S. (2006). Propargyl glycosides as stable glycosyl donors: Anomeric activation and glycosyl syntheses. J. Am. Chem. Soc..

[61] Steber H.B., Singh Y., Demchenko A.V. (2021). Bismuth(III) triflate as a novel and efficient activator for glycosyl halides. Org. Biomol. Chem..

[62] Stévenin A., Boyer F.-D., Beau J.-M. (2010). Regioselective Control in the Oxidative Cleavage of 4,6-O-Benzylidene Acetals of Glycopyranosides by Dimethyldioxirane. J. Org. Chem..

[63] Escopy S., Singh Y., Stine K.J., Demchenko A.V. (2021). A Streamlined regenerative glycosylation reaction: Direct, acid-free activation of thioglycosides. Chem. Eur. J..

[64] Van Steijn A.M.P., Kamerling J.P., Vliegenthart J.F.G. (1992). Synthesis of trisaccharide methyl glycosides related to fragments of the capsular polysaccharide of Streptococcus pneumoniae type 18C. Carbohydr. Res..

[65] Shrestha G., Kashiwagi G.A., Stine K.J., Demchenko A.V. (2022). Streamlined access to carbohydrate building blocks: Methyl 2,4,6-tri-O-benzyl-alpha-d-glucopyranoside. Carbohydr. Res..

[66] Rodebaugh R., Debenham J.S., Fraser-Reid B., Snyder J.P. (1999). Bromination of alkenyl glycosides with copper (II) bromide and lithium bromide: Synthesis, mechanism, and DFT calculations. J. Org. Chem..

[67] Aslanidis P., Cox P.J., Divanidis S., Tsipis A.C. (2002). Copper(I) halide complexes with 1,3-propanebis(diphenylphosphine) and heterocyclic thione ligands: Crystal and electronic structures (DFT) of [CuCl(pymtH)(dppp)], [CuBr(pymtH)(dppp)], and [Cu(m-I)(dppp)]2. Inorg. Chem..

[68] Wittmann V., Lennartz D. (2002). Copper(II)-mediated activation of sugar oxazolines: Mild and efficient synthesis of b-glycosides of N-acetylglucosamine. Eur. J. Org. Chem..

[69] Matsumura Y., Maki T., Murakami S., Onomura O. (2003). Copper ion-induced activation and asymmetric benzoylation of 1,2-diols: Kinetic chiral molecular recognition. J. Am. Chem. Soc..

[70] Tran A.-T., Jones R.A., Pastor J., Boisson J., Smith N., Galan M.C. (2011). Copper(II) Triflate: A Versatile Catalyst for the One-Pot Preparation of Orthogonally Protected Glycosides. Adv. Synth. Catal..

[71] Zhang Z., Wang F., Mu X., Chen P., Liu G. (2013). Copper-catalyzed regioselective fluorination of allylic halides. Angew. Chem. Int. Ed..

[72] Chen I.H., Kou K.G.M., Le D.N., Rathbun C.M., Dong V.M. (2014). Recognition and Site-Selective Transformation of Monosaccharides by Using Copper(II) Catalysis. Chem. Eur. J..

[73] Ahn J.M., Ratani T.S., Hannoun K.I., Fu G.C., Peters J.C. (2017). Photoinduced, copper-catalyzed alkylation of amines: A mechanistic study of the cross-coupling of carbazole with alkyl bromides. J. Am. Chem. Soc..

[74] Ke M., Song Q. (2017). Copper/B2pin2-catalyzed C-H difluoroacetylation-cycloamidation of anilines leading to the formation of 3,3-difluoro-2-oxindoles. Chem. Commun..

[75] Yu F., Dickson J.L., Loka R.S., Xu H., Schaugaard R.N., Schlegel H.B., Luo L., Nguyen H.M. (2020). Diastereoselective sp3 CO bond formation via visible light-Induced, copper-catalyzed cross couplings of glycosyl bromides with aliphatic alcohols. ACS Catal..

