# Supplementary material for: Activation of Thioglycosides with Copper(II) Bromide
Source: Molecules. 2022 Oct 29;27(21):7354. doi: 10.3390/molecules27217354 (PMC9654402; doi:10.3390/molecules27217354)
Supplement: Supplementary file 1 [file molecules-27-07354-s001.zip › molecules-1889096-supplementary.pdf]

Supporting Information

Activation of Thioglycosides with Copper(II) Bromide

Faranak Pooladian,[a] Samira Escopy,[a,b] and Alexei V. Demchenko[a,b]*

[a] Department of Chemistry, Saint Louis University, 3501 Laclede Ave, St. Louis, Missouri 63103, USA;

[b] Department of Chemistry and Biochemistry, University of Missouri – St. Louis, One University Boulevard,St. Louis, Missouri 63121, USA

Contents:

S1. NMR Monitoring to understand mechanistic aspects of the approach S2S2. ^{1}H NMR spectra of known compounds S4

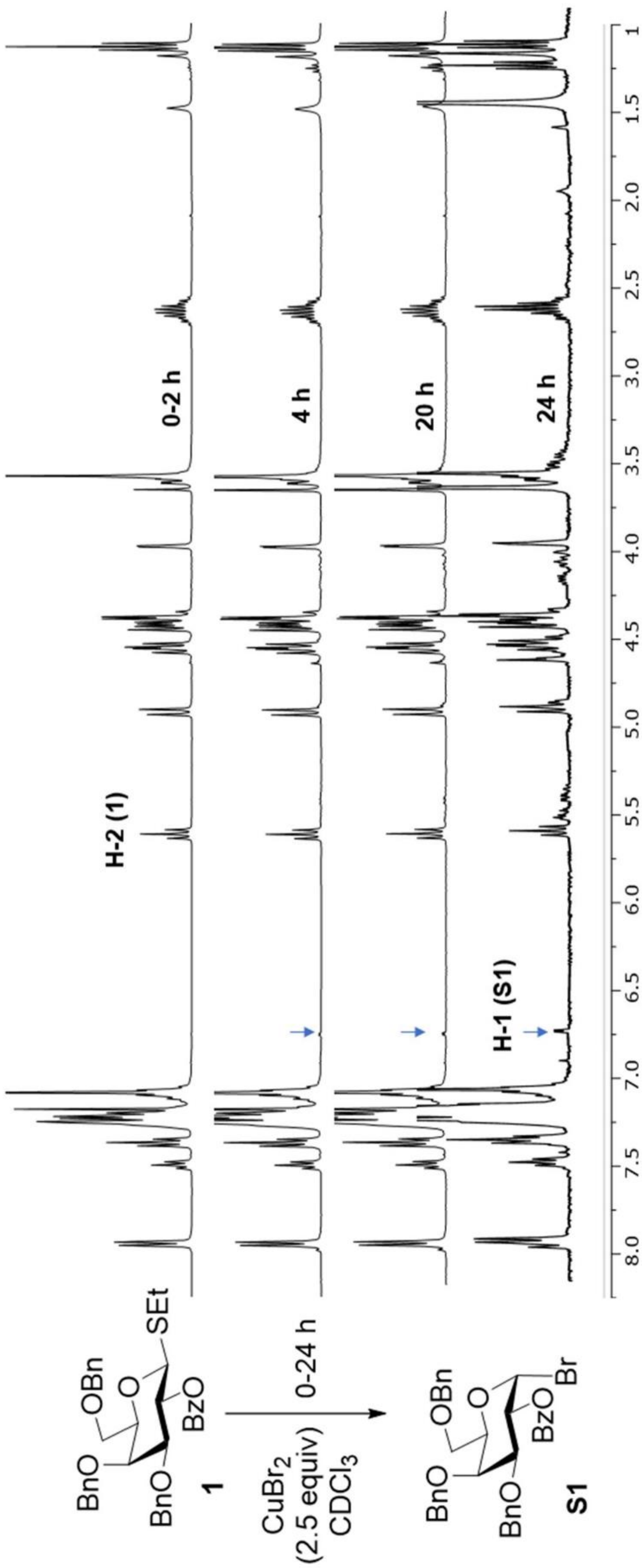

Figure S1: NMR Monitoring to Understand Mechanistic Aspects of the Reaction.

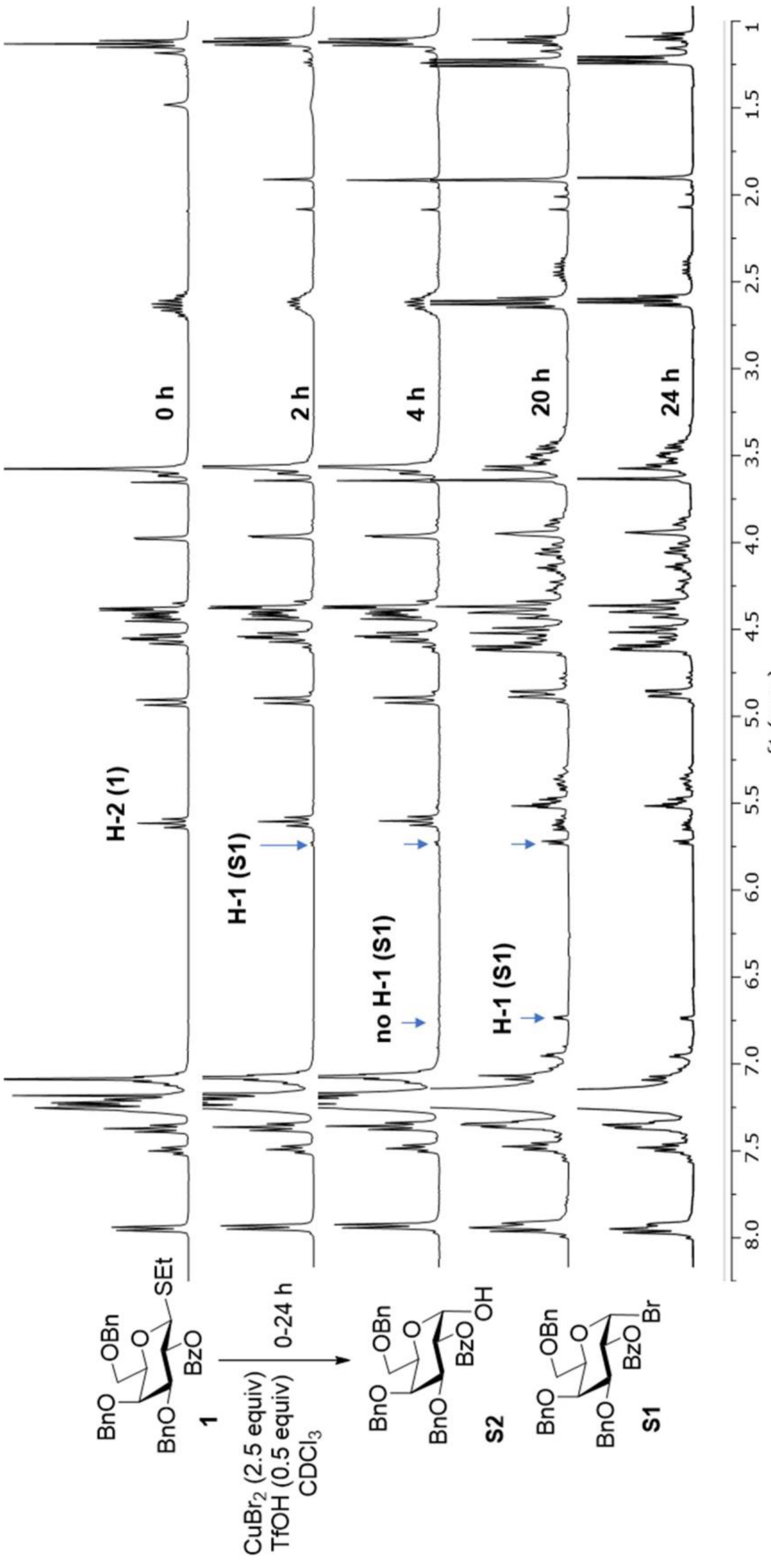

Figure S2: ^{1}H NMR Sectra of Known Compounds.

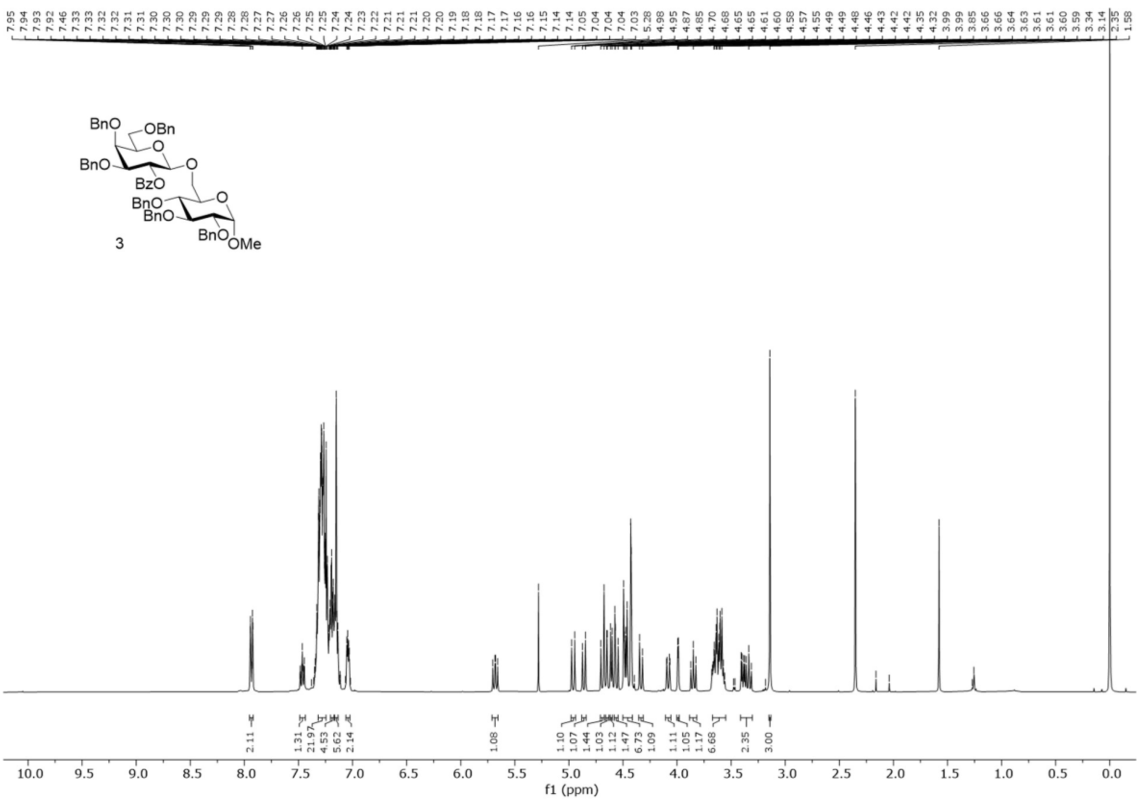

Figure S3: ^{1}H NMR (CDCl3 400 MHz) of compound **3**.

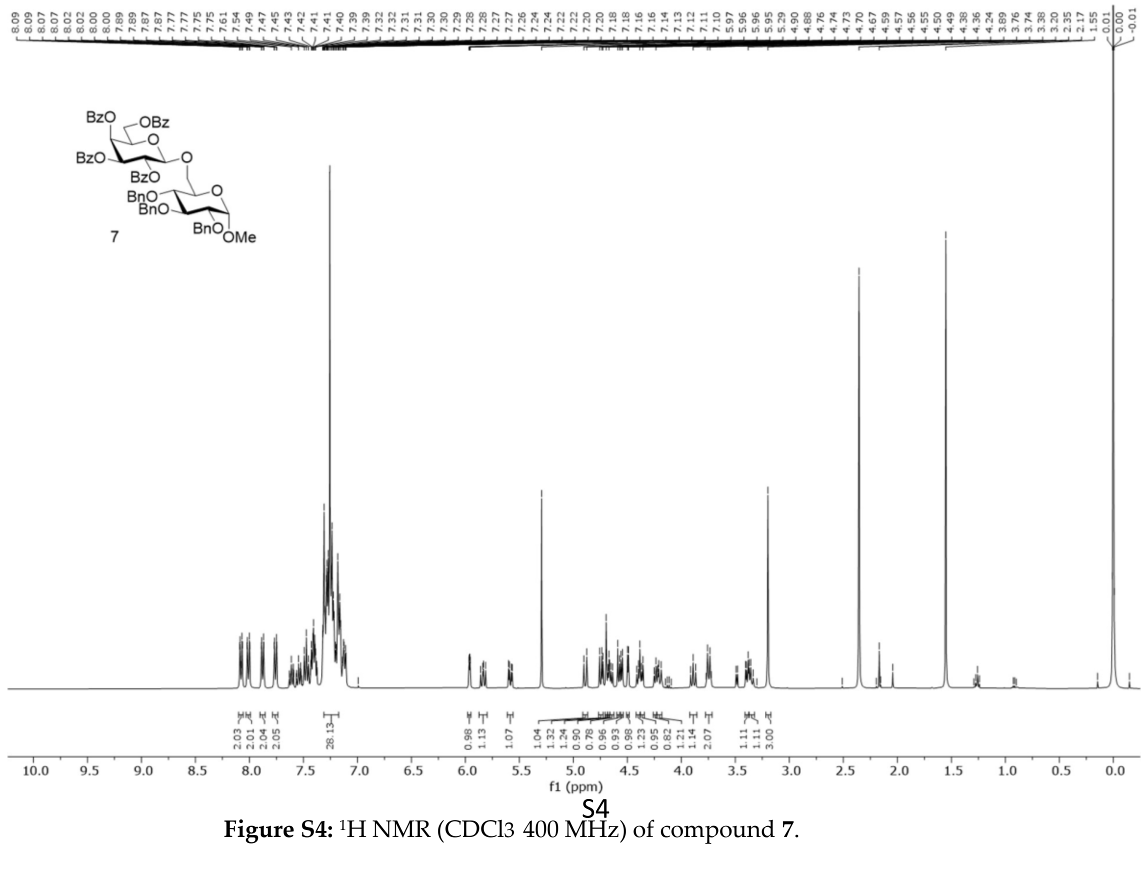

Figure S4: ^{1}H NMR (CDCl3 400 MHz) of compound **7**.

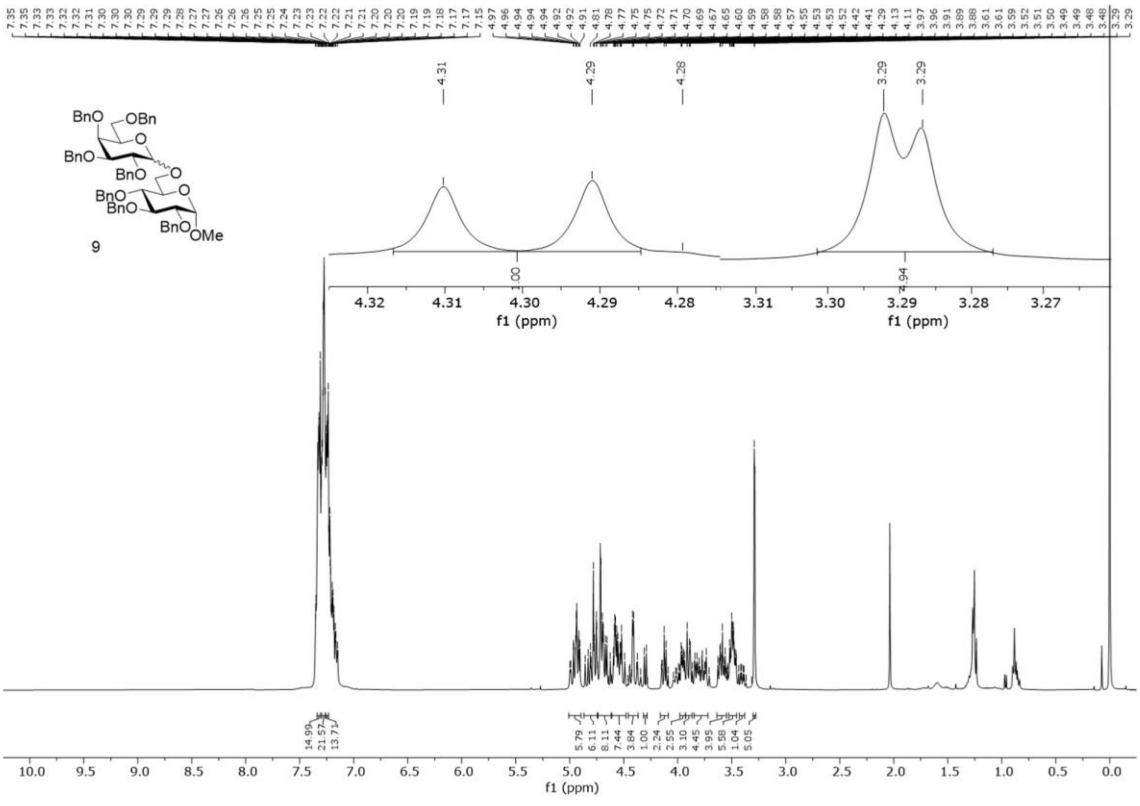

9

Figure S5: ^{1}H NMR (CDCl3 400 MHz) of compound **9.**

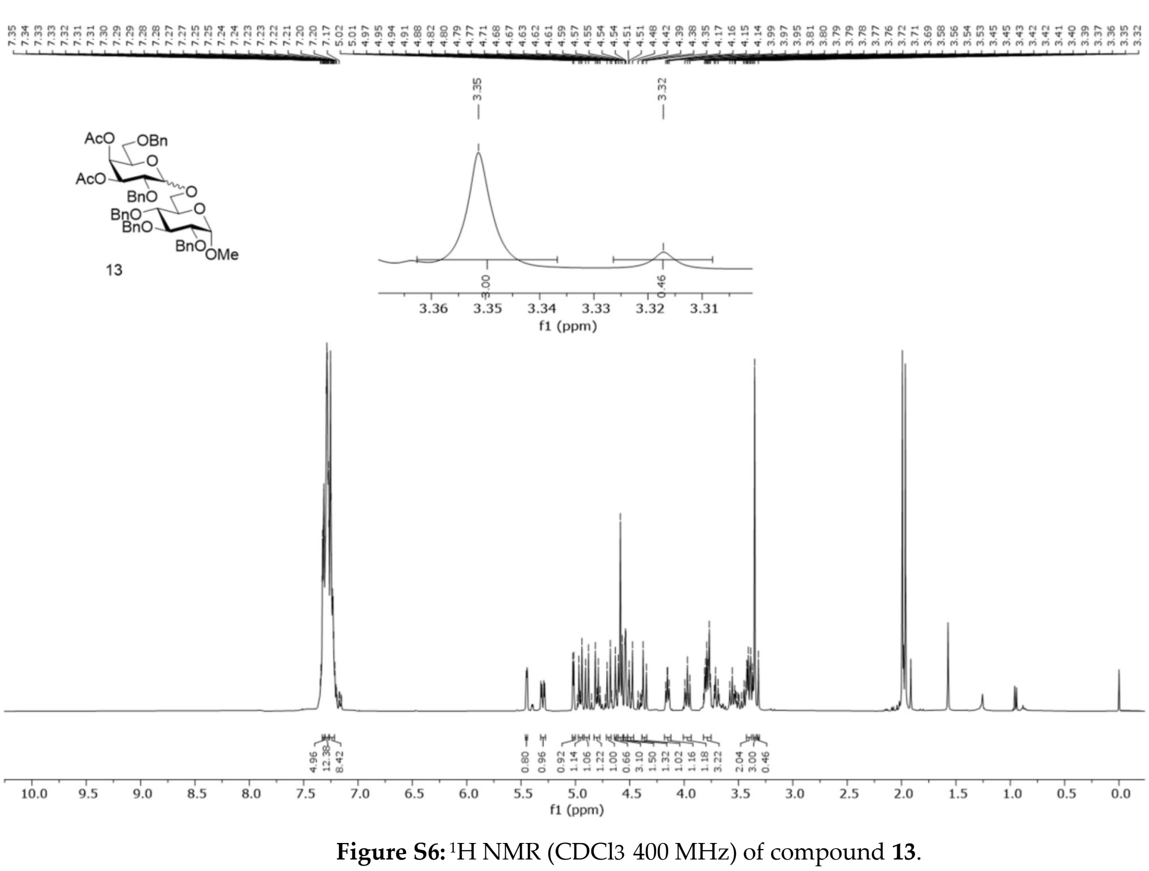

13

Figure S6: ^{1}H NMR (CDCl3 400 MHz) of compound **13**.

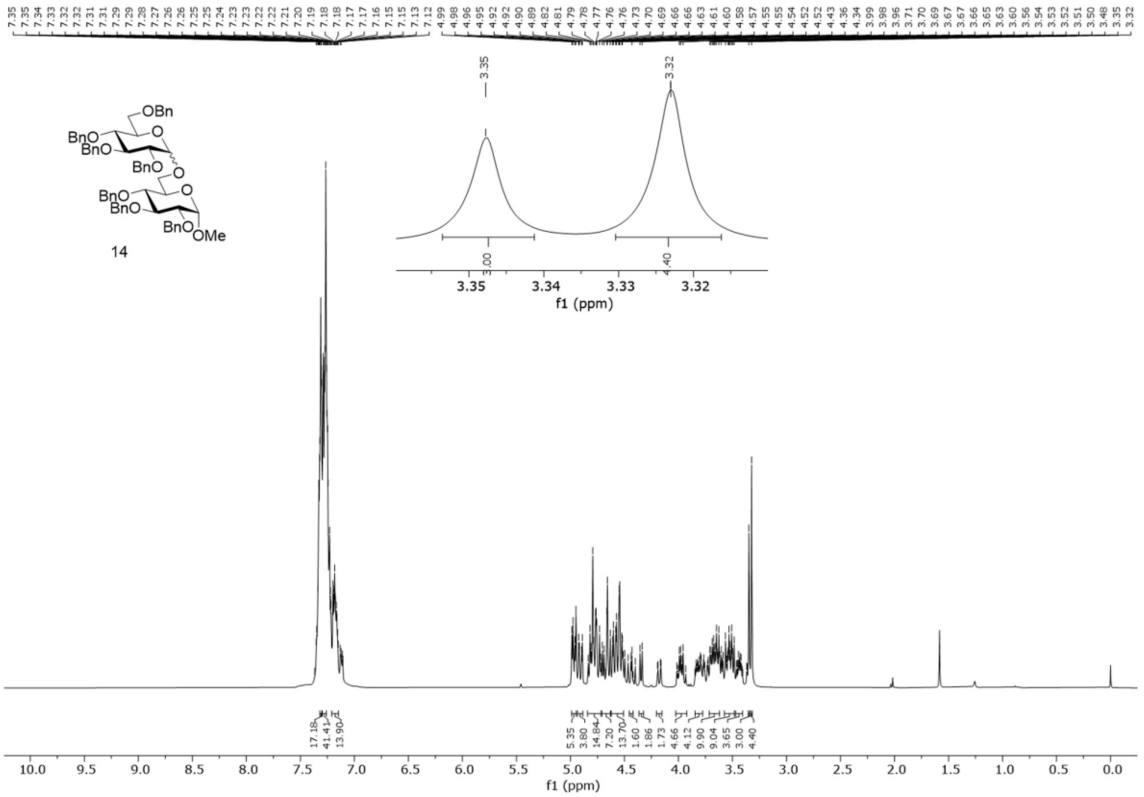

Figure S7: ^{1}H NMR (CDCl3 400 MHz) of compound **14**.

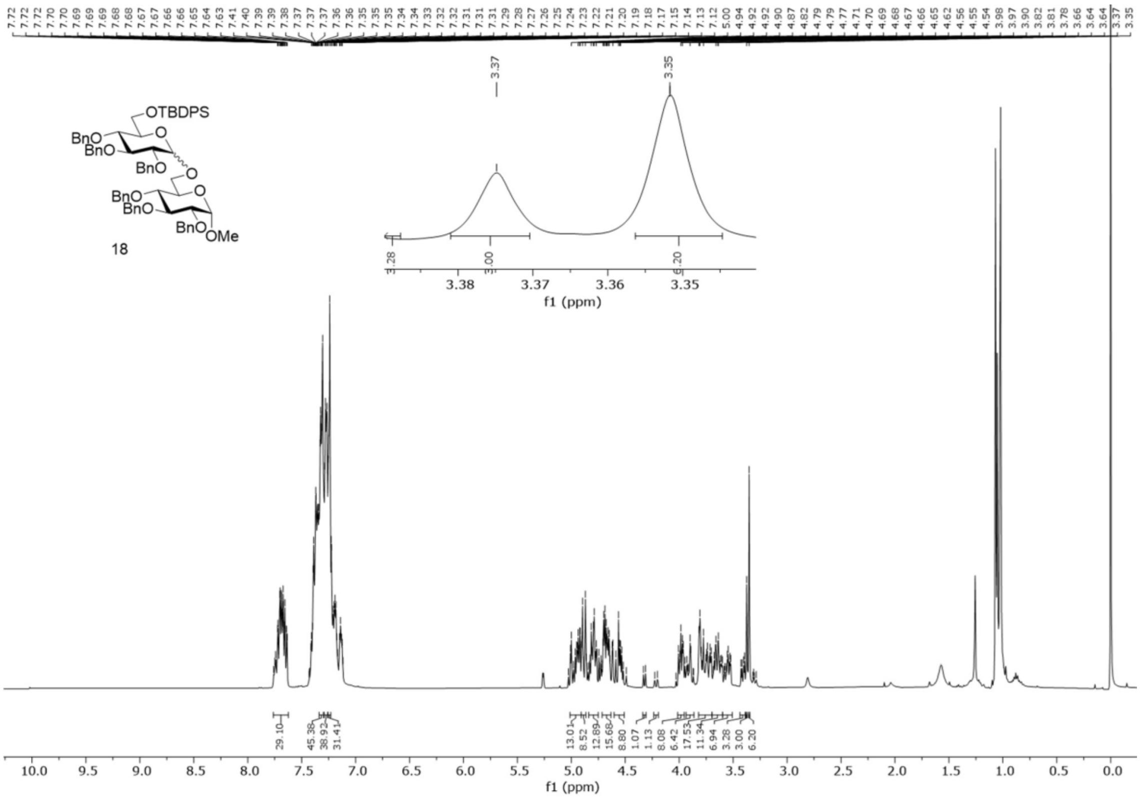

Figure S8: ^{1}H NMR (CDCl3 400 MHz) of compound **18**.

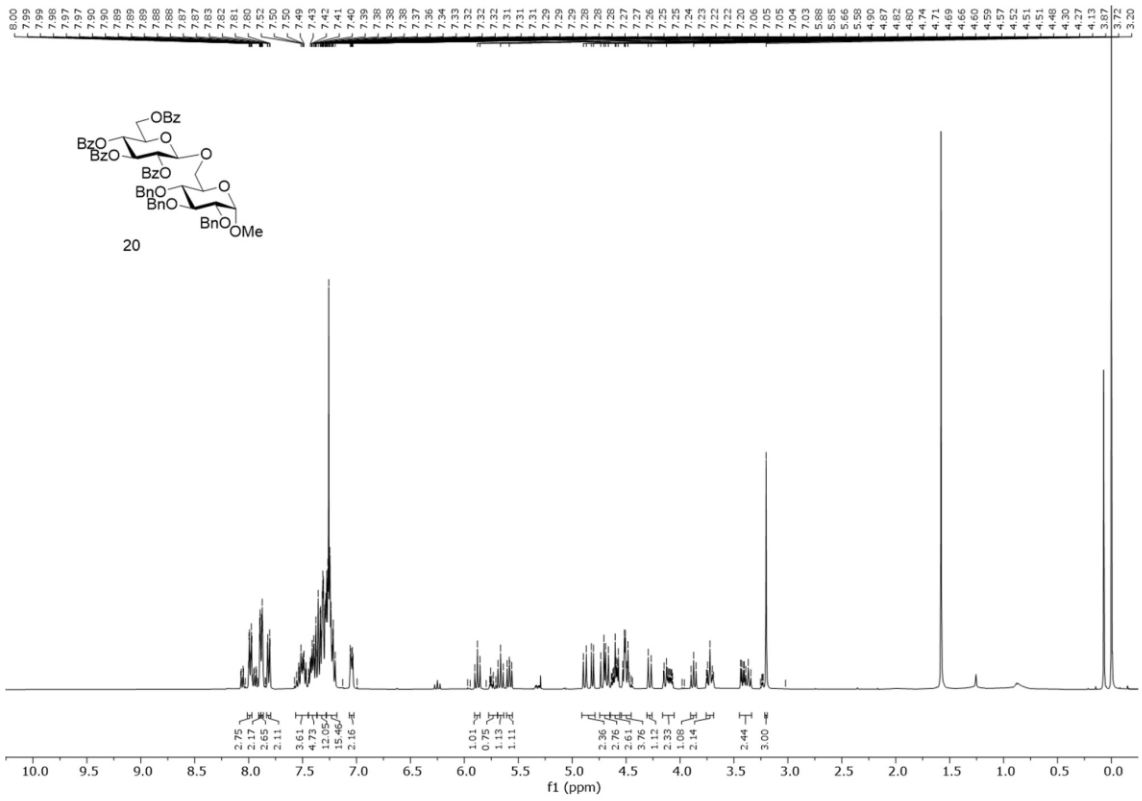

Figure S9: ^{1}H NMR (CDCl3 400 MHz) of compound **20**.

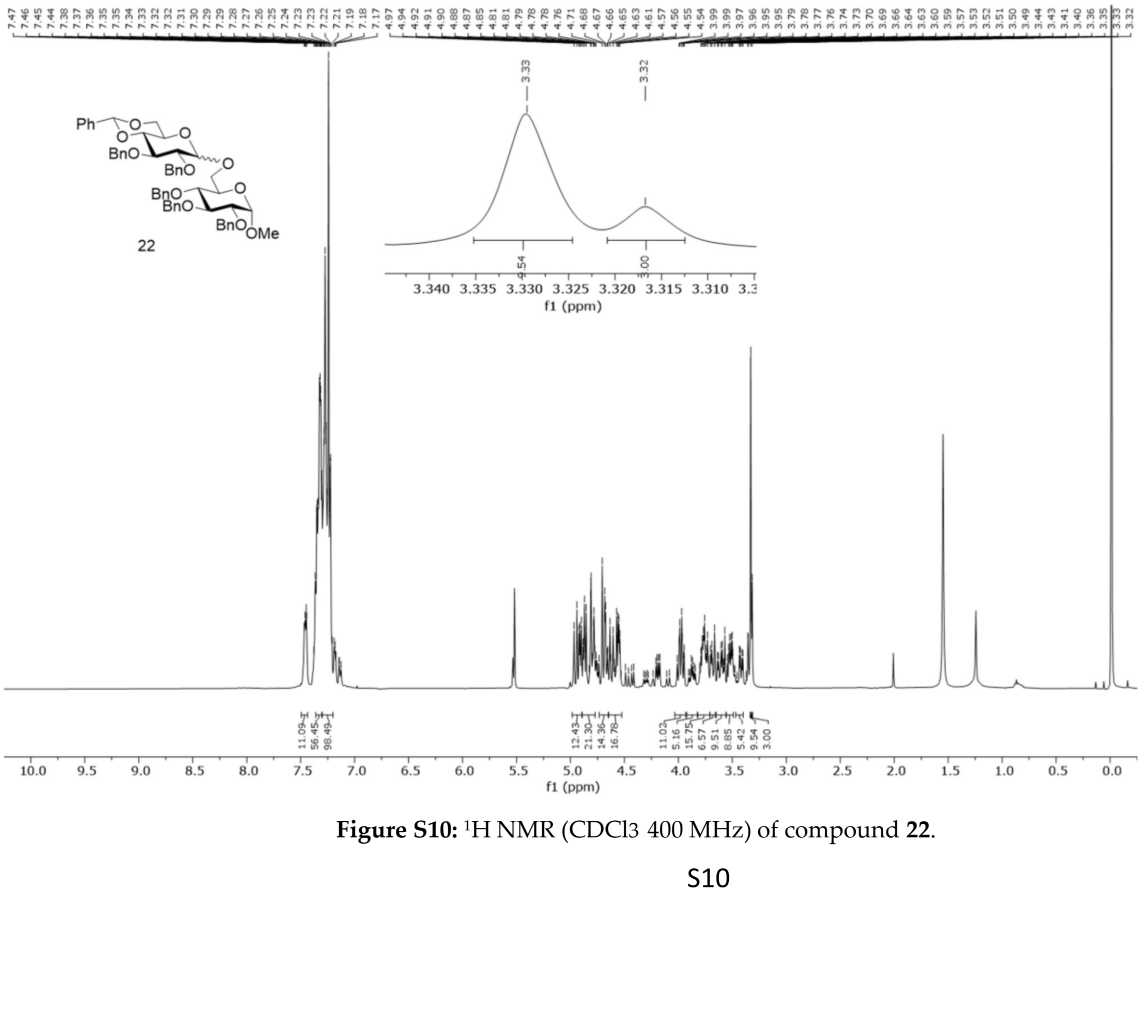

Figure S10: ^{1}H NMR (CDCl3 400 MHz) of compound **22**.

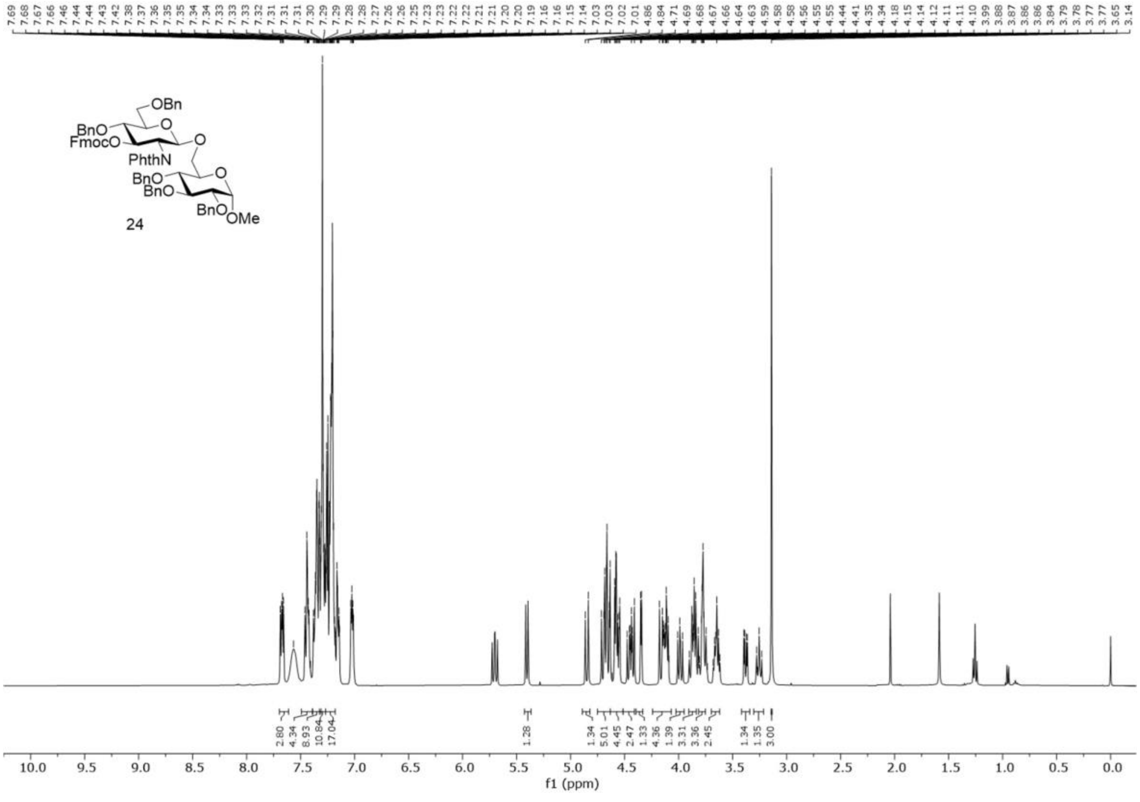

Figure S11: ^{1}H NMR (CDCl3 400 MHz) of compound **24**.

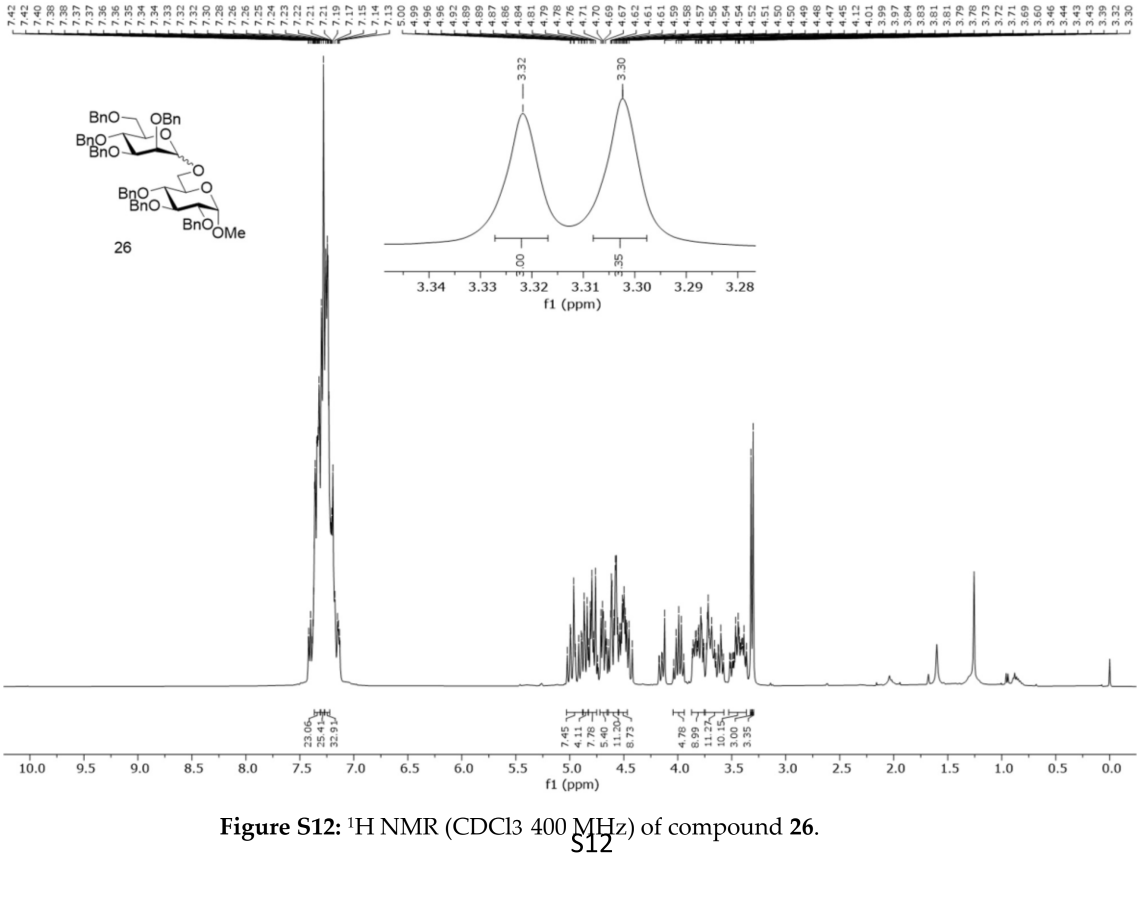

Figure S12: ^{1}H NMR (CDCl3 400 MHz) of compound **26**.

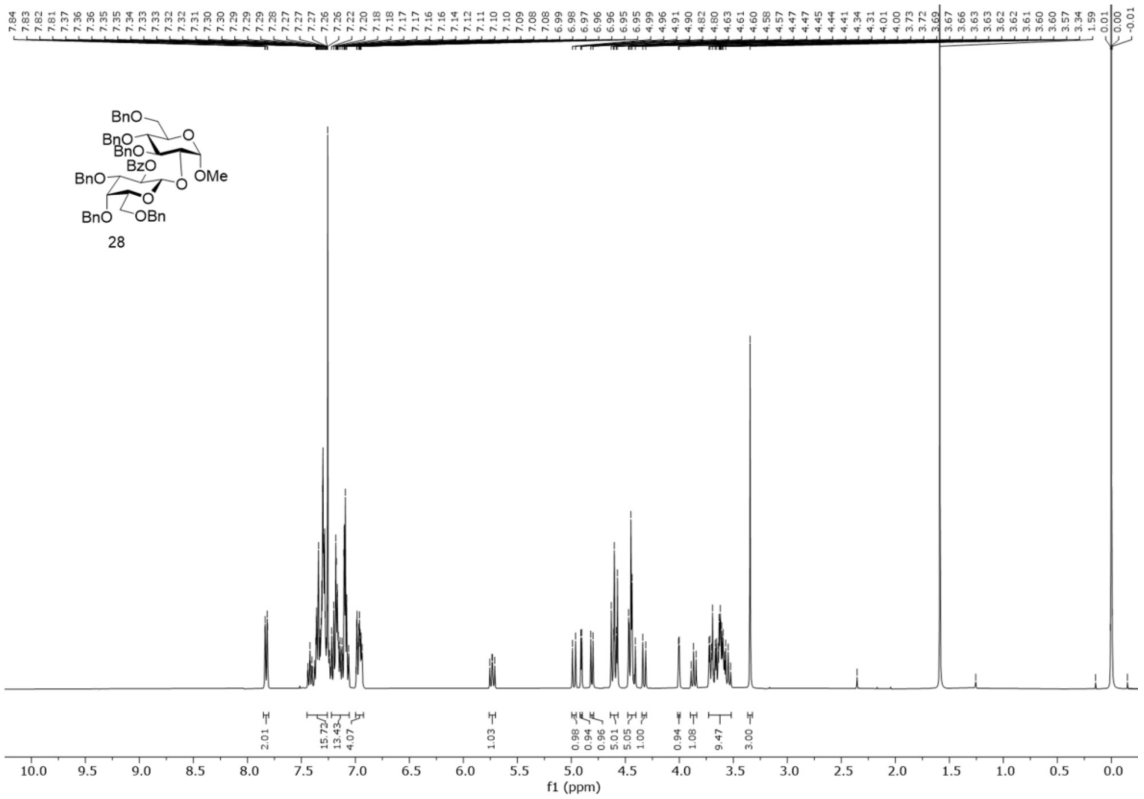

Figure S13: ^{1}H NMR (CDCl3 400 MHz) of compound **28**.

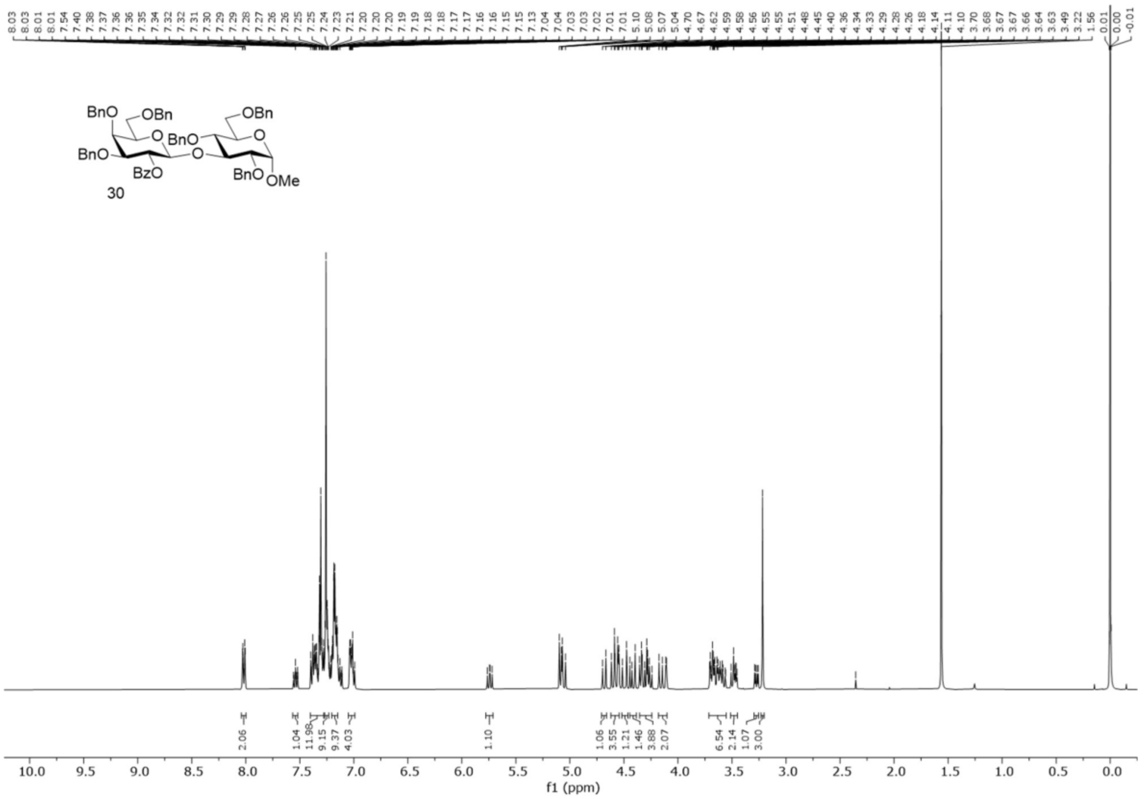

Figure S14: ^{1}H NMR (CDCl3 400 MHz) of compound **30**.

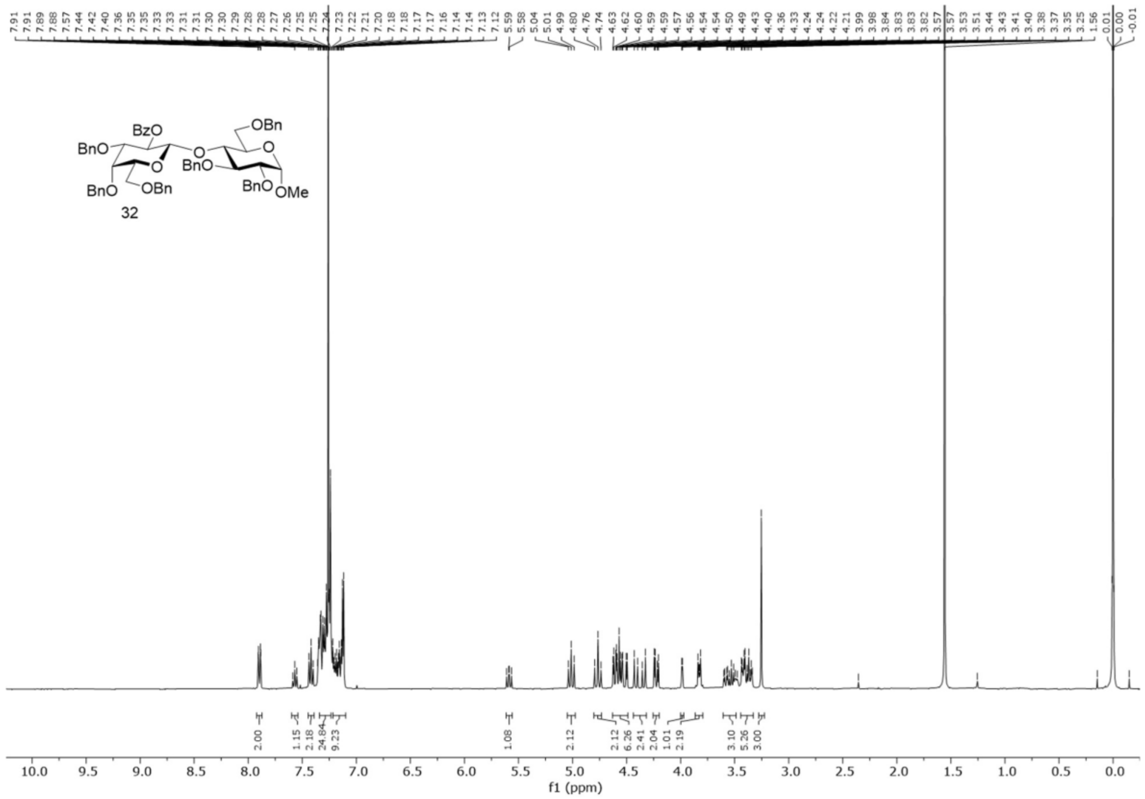

Figure S15: ^{1}H NMR (CDCl3 400 MHz) of compound **32**.

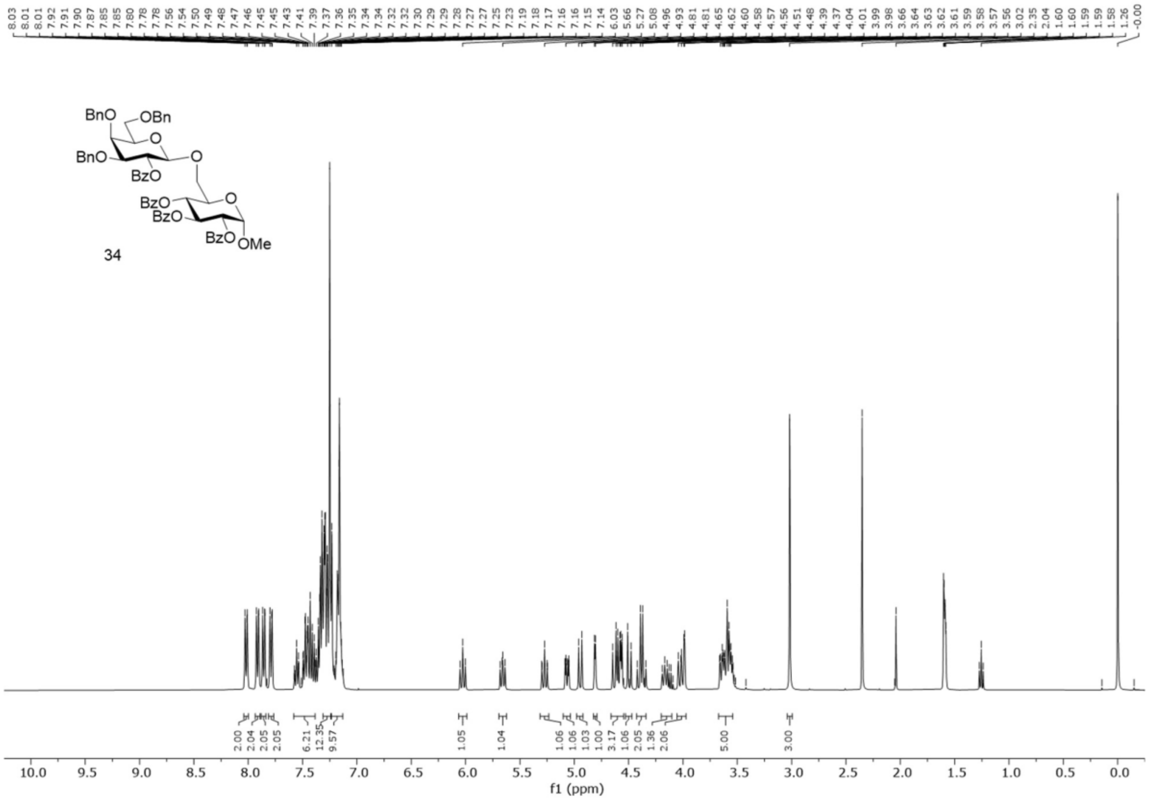

Figure S16: ^{1}H NMR (CDCl3 400 MHz) of compound **34**.
